# Draft Genome Sequence of Bacillus toyonensis Strain GM18, Isolated from Agricultural Soil

**DOI:** 10.1128/MRA.00008-21

**Published:** 2021-03-04

**Authors:** Carlos J. Moreno-Peña, Jorge H. García-García, Cesar I. Hernández-Vásquez, Luis J. Galán-Wong, Benito Pereyra-Alférez

**Affiliations:** a Instituto de Biotecnología, Facultad de Ciencias Biológicas, Universidad Autónoma de Nuevo León, San Nicolas de los Garza, Nuevo León, Mexico; Georgia Institute of Technology

## Abstract

Bacillus toyonensis is a recently described species related to Bacillus cereus and Bacillus thuringiensis. The GM18 strain previously identified as B. thuringiensis is now classified as *B. toyonensis* based on the RNA 16S sequence and whole-genome average nucleotide identity. The genome analysis revealed the presence of insecticide, nematicide, and antitumoral proteins.

## ANNOUNCEMENT

The GM18 bacterium was isolated from agricultural soil collected in the state of Guanajuato, Mexico, using the methodology described by Travers et al. (1), and based on its H antigen characteristics, this strain was recognized as a new serotype (H-24) and named Bacillus thuringiensis subsp. *neolonensis*. In an early description, Rodriguez-Padilla et al. (2) showed that GM18 possesses characteristics of the B. thuringiensis group; it is Gram positive and sporulated and synthesizes a parasporal crystal. However, it had no insecticidal activity. Later, when the inclusion body was tested against cancer cell lines, solubilized proteins displayed biological activity, and cytotoxicity ranged from low against 1c1c7 and A431 to high against A375 and BpRc1 cell lines, but no cytotoxicity for sheep erythrocytes was detected (3).

For the genome sequencing of *B. toyonensis* GM18, cells obtained from our laboratory cryostock were grown in 25 ml of LB broth for 6 h at 30°C, and then the pellet was recovered after centrifugation and washed with sterile water. The pellet was shipped chilled to Genewiz (New Jersey, USA), where total DNA extraction was performed, and the DNA library was prepared with the Illumina DNA prep, (M) tagmentation, and IDT for Illumina DNA/RNA indexes set A tagmentation kits (Illumina, Inc., California, USA). Whole-genome sequencing of *B. toyonensis* GM18 was performed with paired-end Illumina MiSeq sequencing, which resulted in 11,175,537 paired-end reads of 137 to 150 bp with a coverage of 250×; for genome assembly and annotation, we used Galaxy (4). In brief, the sequence quality was determined with FastQC (Galaxy version 0.72+galaxy1) (5). Then, low-quality sequences and adapters were removed with Trimmomatic (Galaxy version 0.038.0) (6). The processed sequence reads were assembled with the Shovill pipeline, including the SPAdes assembler (Galaxy version 1.1.0+galaxy0) (7); the resulting contigs were annotated with Prokka (Galaxy version 1.14.5+galaxy0) (8) and with the NCBI Prokaryotic Genome Annotation Pipeline (PGAP) using the best-placed reference protein set method (GeneMarkS-2+) (9, 10); 91.59% of all sequences were assembled in 156 contigs with an *N*_50_ value of 171,900 bp. Default parameters were used for all software.

We found that GM18 has a 6.13-Mb genome containing 6,129 open reading frames (ORF) with a GC content of 34.93%. The genome has 96 operons for tRNAs and 16 for rRNAs. The average nucleotide identity (ANI) of the whole genome determined during the GenBank submission process (11) showed 99.018% identity with the type genome of Bacillus toyonensis; this and the sequence identity of the rRNA 16S (GenBank accession number JADNYS010000095.1), *gyrB* (MBF7148837.1), *groEL* (MBF7150603.1), and *XRE* (MBF7150840.1) genes suggests that this isolate belongs to the *B. toyonensis* species (12).

In brief, the sequence of the GM18 genome could be key to understanding the *Bacillus* group and its members’ evolutionary relations. This genome shows greater identity with Bacillus toyonensis, although it has protein-coding genes with identity to cry (GenBank accession number WP_195755641.1, WP_195755715.1), vip (WP_195755724.1, WP_195755747.1), and parasporin (WP_195755716.1) that are characteristic of B. thuringiensis species; it also has insertion sequences with identity to those of B. thuringiensis, but this could be because *B. toyonensis* is a relatively newly described species of the *Bacillus* group (13).

### Data availability.

The Bacillus toyonensis GM18 whole-genome shotgun sequence has been deposited in GenBank under the accession number JADNYS000000000 and BioProject number PRJNA676032. The raw sequence files can be found under SRA number SRP301246.
